# From child-peer similarity in imitative behavior to matched peer-mediated interventions in autism

**DOI:** 10.3389/fpsyg.2023.1173627

**Published:** 2023-08-02

**Authors:** Jean Xavier, Simona Johnson, David Cohen

**Affiliations:** ^1^Department of Child and Adolescent Psychiatry, Henri Laborit Hospital Centre, Poitiers, France; ^2^CNRS UMR 7295, Équipe CoCliCo, Cognition and Learning Research Center, Poitiers, France; ^3^Faculty of Medicine and Pharmacy, University of Poitiers, Poitiers, France; ^4^Department of Child and Adolescent Psychiatry, Reference Centre for Rare Psychiatric Diseases, AP-HP, Groupe Hospitalier Pitié-Salpêtrière, Sorbonne Université, Paris, France; ^5^CNRS UMR 7222, Institute for Intelligent Systems and Robotics, Sorbonne Université, Paris, France

**Keywords:** autism, interpersonal affordances, imitation, similarity, peer-mediated interventions, self and others affordance

## Abstract

Self-consciousness develops through a long process, from pre-reflexive consciousness relying on body perception, to “meta” self-awareness. It emerges from the imitative experience between children and their peers. This experience linked to the capacity to test structural similarities between oneself and others, is addressed according to the concept of interpersonal affordance. We hypothesize that the opportunity for co-actors to engage in a process of interpersonal coordination is underlined by their similarity in terms of morphological, behavioral and motor features. This experience can sustain the emergence of new affordances for objects for each co-actor, as well as new affordances in terms of joint actions. We apply this idea in the context of peer-mediated interventions (PMI) in autism spectrum disorder (ASD). We argue that, in PMI, an encounter between children with autism and similar peers would foster the opportunity to engage in a spontaneous process of interpersonal coordination. This process would enable the development of self-consciousness and the emergence of perception of interpersonal, self and other’s affordances for children with autism. We conclude that metrics to assess morphological, behavioral and motor similarity should then be defined and used in future studies to test our hypothesis in children with autism versus TD children or between children with autism.

## 1. Introduction

In a developmental perspective, cognition is shaped by the body and its sensory motor interactions, arising in real time between children and their world.

Self-consciousness allows both self-recognition and self–other differentiation and is the basis of social interactions. It develops through a long process, from pre-reflexive consciousness relying on body perception to “meta” self-awareness (Keromnes et al., 2019). Self-consciousness emerges from the imitative experience between children and their peers (Xavier et al., 2016).

Imitation plays a major role in the development of intersubjectivity in terms of communication and conceptual understanding of self and other as intentional agents (Tomasello, 1999). According to its two faces, to imitate and recognize being imitated, this shared experience is a primary means of interaction between peers before language becomes firmly established (Eckerman and Didow, 1996). Imitation corresponds to a process of interpersonal coordination. It is linked to the perception that others are “like me” (Meltzoff and Decety, 2003) and to the capacity to test similarities between oneself and others (Nadel, 1986). We address this capacity according to the concept of interpersonal affordance which corresponds to the fit between self and other body properties (Gibson, 1979).

On the basis of the notion of similarity we hypothesize that, in a child-peer encounter, the opportunity to engage in a motor imitation process is fostered by a situation of interpersonal affordance. This situation involves their similarity in terms of (1) morphological (physical resemblance, size, geometric ratios), (2) behavioral (style of actions) and (3) motor features (kinematics, core geometric patterns of invariants biomechanical ratios).

Moreover, considering the relationship between interpersonal affordance and affordances for objects, during imitative experience the former could direct the perception of the latter, i.e., could direct the perception of self and other’s affordances.

Finally, this experience could also enable the emergence of new action possibilities, which extend the affordances boundaries that are possible for each co-actor, toward the development of joint actions.

We apply this idea in the context of peer-mediated interventions (PMI) in autism spectrum disorder (ASD). This neurodevelopmental condition is characterized by developmental deficits in social communication. It also includes self-consciousness impairments associated with problems in motor imitation in its two faces, imitation production and imitation recognition.

In PMI, TD peers helping children with ASD are chosen by therapists for their strong social skills and educated using peer strategies. Even if PMI are considered as an interesting approach to address social communication deficits for children with ASD, their efficacy remains underexamined (Chang and Locke, 2016).

In line with our ‘similarity hypothesis’, some interventions for children with ASD involve the imitation of the child’s actions by therapists or parents, as a strategy to promote social engagement (for a review see Contaldo et al., 2016). These behavioral interventions are mainly used in children at an early age or with low developmental and imitation abilities. Contaldo et al. (2016) conclude that the behavioral similarity between the actions of children and the following action of an adult is an effective strategy to promote social engagement and generalized imitation in this population.

However, the generalization of imitative behaviors were not assessed in the children’s natural environment or during play with similar aged peers. Furthermore, mirroring or rhythm are used in Dance and Movement Therapy (DMT), involving therapist-child interactions, in increasing communication and social skills in children with ASD (Mastrominico et al., 2018).

Our general hypothesis is that child-peer similarity fosters ‘imitability’ and, consequently, facilitates subsequent cognitive processing including inhibition abilities. We argue that: for children with autism, particularly those with lower cognitive functioning, encounter between similar peers, either TD children or children with autism, promotes the opportunity to engage in imitative exchanges. The contiguity between oneself and others, through a proprioceptive and kinesthesic matching, could facilitate behavioral mirroring and interpersonal synchronization.

Such a tailored environment, through behavior copying, provides the conditions for a spontaneous dynamic interpersonal coordination process leading to the development of self-consciousness and the emergence of perception of interpersonal, self and others’ affordances. During child development, the continuous congruence between one’s own body size and motor ability evolution, and that of others, would sustain a generalization of peer-interactive experiences favoring adaptation for children with autism.

We argue that, in PMI, an encounter between children with autism and similar peers would promote the opportunity to engage in a spontaneous process of interpersonal coordination. This process would enable the development of self-consciousness and the emergence of the perception of interpersonal, self and other’s affordances for children with autism. These interventions could involve typically children with autism with TD children, or only children with autism. During development, given physical growth and evolution of motor skills, this process would sustain a generalization of peer-interactive experiences and further, for children with autism, allow better adaptation to the environment.

## 2. Peer imitation in children: a dynamic embodied process of interpersonal coordination

Humans have a naturally developed propensity to attune to one another (Kronsted and Gallagher, 2021). Attunement is ‘a kinesthetic and emotional sensing of others knowing their rhythm, affect and experience by metaphorically being in their skin, and create a two-person experience of connectedness’ (Erksine, 1998). Gaze following, body posture and gesture synch up in social interactions, giving us access to intentions and emotions of others (Kronsted and Gallagher, 2021). From a developmental perspective, self-consciousness considered as a process, emerges during the child’s interactive experience with his environment allowing comparison and distinction between oneself and others (Keromnes et al., 2019).

The emergence of self-other distinction is at the heart of the imitative process (de Waal, 2008). Imitation is critical to the development of human intersubjectivity (Nadel, 2002; Meltzoff and Decety, 2003). According to its two faces, imitate and being imitated, it provides the sense of a shared experience involving ‘social mirroring, social modeling and self-practice’. Imitation is a prerequisite of the self (Meltzoff, 1990). From infancy, ‘being imitated’ promotes a social orientation toward others (Nadel, 2002; Agnetta and Rochat, 2004). After being imitated, infants engage in “testing behaviors” (i.e., repeating or varying actions while watching the imitative partner) to test whether the other is imitating them (Nielsen, 2006). Motor imitation is a shared experience with the ability to integrate others into one’s own perspective including the capacity to switch from an ego- to an heterocentered perspective (Xavier et al., 2016).

Explored through a developmental lens, spontaneous child-peer motor imitation corresponds to a process of interpersonal coordination which includes two main components (Xavier et al., 2013):

- Behavioral matching or spontaneous mimicry, defined as the tendency to reproduce the low-level kinematic features of any modeled action. This social mimesis includes mirroring positions, similarity in bodily actions and postural congruence (Tanaka, 2015), but not a perfect replication of another’s posture or behavior.
- Interactional synchrony, referring to similar rhythmic movement between partners creating smooth coordination (Trees, 2009). It also refers to the degree of congruence between the behavioral cycles of engagement and disengagement of the partners (Leclère et al., 2014). Interpersonal synchronization also exists at the submovements level, which make up motor control (Tomassini et al., 2022). Child-peer interpersonal synchronization enhances a sense of similarity in movement and posture through synergistic perception–action systems (Marsh et al., 2006).

Wallon (1949) describes the ‘autoplastic imitation’ period, during the third year of life, when the child spontaneously reproduces by modeling his own body, the character of the other, his postures, gestures and attitudes. For example, when a child sees a peer who starts running, spontaneously he or she will do the same thing without being aware of his peer intention. From this period of relative self-other indistinction, imitative exchanges gradually lead to a situation of role reversal and reciprocity, whereby each partner is able to identify his or her own purpose and intentions (Wallon, 1956). Imitation is a process that establish both psychological connectedness and differentiation between ‘self’ and ‘other’.

During this process, partners are mutually co-constituted, in terms of self-consciousness and self-agency, by smoothly adapting to each other’s ongoing actions within the interaction they tend to sustain (Kimmel et al., 2018). Nadel-Brulfert and Baudonniere (1982) studied spontaneous imitation in two-year-old peers. They found that the use of identical objects and similar posture between peers favored instigation and pursuit of interaction. Imitation and imitation recognition are tightly linked as two facets of the same innate capacity to test structural similarities between oneself and others (Nadel, 1986).

This shared experience is underlined by the perception that others are ‘like me’ (Meltzoff, 2007) through the existence of a structural congruence between the perception of others and one’s own behavior (Meltzoff and Decety, 2003).

We address this similarity between oneself and others through the concept of interpersonal affordance.

## 3. Child-peer interpersonal coordination: a developmental experience of interpersonal affordance

Perceiving others is not passively receiving the stimuli, but actively seeking potential actions and taking up the same action as them (Varela et al., 1991). In line with the ecological psychology theory, the attunement between partners relies on the suitable match between each of their body properties. Affordances are co-constituted in the agent-environment relationship, defined by the fit between environmental features perceived by the senses and personal features (bodily scales and dimensions, action capabilities, postural and kinematic aspects; Dijk and Rietveld, 2017).

Numerous studies show that people are able to perceive self-affordances (Warren and Whang, 1987; Mark et al., 1997; Creem-Regehr et al., 2013) grounded in an egocentric point of view and scaled in terms of one’s body dimensions. Younger children make more errors than elders and adults in estimation for reaching, stepping and ducking. Franchak (2019) compared the ability of children and adults to decide whether they could squeeze through doorways of varying width. They found that judgment accuracy improved with age and that participants had more difficulty when they had to recalibrate their perception of action possibilities to account for sudden changes to body size.

To determine others’ affordances it is necessary, taking an heterocentered perspective, to scale the environment to the other’s body capabilities (Stoffregen et al., 1999).

Rochat (1995) examined reaching affordances of children and adults, asking whether young children could distinguish reachability for themselves from an actor’s. The findings revealed that both children and adults could; however, children’s judgments tended to be more inaccurate than adults’, underestimating the actor’s reaching ability. Furthermore, perceivers can also use dynamic information about others’ physical abilities to form judgments of another’s action capabilities.

In actions like jumping-and-reaching, several studies (for a review see Gagnon et al., 2020) revealed that:

- Having access to visual information about the actor’s kinematic abilities allows more accurate prediction of their action boundaries, and
- Experiencing these actions improves the accuracy of these predictions.

Others are for humans the ‘richest and most elaborate affordances of the environment’ (Gibson, 1979). They constitute salient features of the world as they attract a disproportionate percentage of human perceptual attention (Fletcher-Watson et al., 2008). Social affordances are perceptions of opportunities for coordination, directly perceived and arising from interaction (Steffensen, 2013).

Costantini et al. (2011) argues that interpersonal affordance is underlined by a space mirror mechanism that allows each partner to match the others’ surrounding space with one’s own peripersonal space, i.e., mapping others’ action potentialities onto one’s own motor abilities. Similarly to motor imitation, this mechanism involves the ability to integrate others into one’s own perspective which presupposes the capacity to switch from an ego- to an heterocentered perspective.

A situation of interpersonal affordance involves the ability of each partner to perceive structural and transformational invariants characterized as “modes of change” over time, as well as dynamic regularities (Warren, 1998). During this real-time coupling between co-actors, action possibilities are selected, modulated and modified according to the evolving interactional contingencies. Thus, the emergence of new action possibilities, in terms of joint actions, extends the affordances boundaries that are possible for each co-actor (Lopresti-Goodman et al., 2009).

According to the concept of emulation (Tomasello, 1990), interpersonal affordance and affordance for objects are intimately linked, the former directing the latter. An observer learns environmental properties in an indirect manner. Seeing another doing an act using some object, in a particular context, increases the observer’s probability of behavior copying, interacting with that object (Byrne, 2009).

Interpersonal affordance during imitative experience could then direct perception of self-affordances and other’s affordances (*CF.* Table 1).

**Table 1:** Definitions.

Peer- imitative experience*		
To imitate and recognize being imitated	Interpersonal coordination	Behavioral matching
		Interpersonal synchrony
Affordances**	In the agent-environment relationship, they are defined by the fit between personal and environmental features (adults, peers, objects, …)	
Self-affordances	Perception of self-possibilities for action on environment, grounded in an egocentric point of view	
Others’ affordances	Perception of others possibilities for action on environment, according to an heterocentered point of view	
Interpersonal affordances	Integration of point of views, including the ability to switch from an ego- to an heterocentered perspective. Directing the perception of self-affordances and promoting the perception of others’ affordances. Enabling the emergence of new action possibilities, extending the affordances boundaries possible for each co-actor.	

*Nadel (2002), Xavier et al. (2016), and **Gibson (1979).

## 4. General hypothesis: child-peer similarity fosters ‘imitability’

On the basis of the notion of similarity, child-peer encounters could offer conditions to promote a situation of interpersonal affordance, i.e., the opportunity for co-actors to engage in a process of interpersonal coordination.

Słowiński et al. (2016) demonstrated the existence of a time-invariant individual motor signature that affects the synchronization level between players performing a joint action task. Every individual moves following a “human-like way of movement” with unique kinematic features (Kilner et al., 2007). In line with Nadel’s studies (mentioned above), similarity is a crucial determinant for social interactions. Starting in infancy, group members are expected to act in similar ways (Powell and Spelke, 2013). Infants expect individuals who display the same ritualistic actions or who exhibit similar preferences to be affiliated (Liberman et al., 2016).

Several studies in social sciences have demonstrated that people are strongly attracted to similar physical and behavioral traits (Bardy et al., 2014). Studies of interpersonal interaction have shown that when two people possess similar movement dynamics they spontaneously coordinate their movements with each other (Romero et al., 2012).

We hypothesize that, in a child-peer encounter, the opportunity to engage in a motor imitation process is fostered by a situation of interpersonal affordance: opportunities for coordination are directly perceived and arise from the encounter between the co-actors. This situation involves similarity in the following features:

- Morphological (physical resemblance, size, geometric ratios)
- Behavioral (style of actions)
- Motor features (kinematics, core geometric patterns of invariants biomechanical ratios).

Furthermore, as said above, this child-peer interactive experience does not rely on a perfect replication of spatial positions, sizes, shapes and synchronization. The delta allows the partners of the interaction to maintain a degree of autonomy and drive the process forward, leading to the emergence of qualitatively new action possibilities: this experience of interpersonal affordance can sustain the emergence of new affordances for objects, for each co-actor, as well as new affordances in terms of joint actions.

This similarity could facilitate the perception of structural and transformational invariants. The contiguity between one’s body and another’s through proprioceptive and kinesthetic-visual matching allows putting oneself in the shoes of somebody else. To some extent, their high degree of congruence in terms of body properties, and the quality of their interactional synchrony refer to a mirror self-experience.

Furthermore, this experience of body perception offers an intermodal calibration of one’s own body, allowing the development of body awareness, i.e., a body schema (Rochat and Zahavi, 2011).

This type of experience reminds us of relationships with different types of doubles: (1) in autoscopic phenomena described by Blanke and Mohr (2005) in patients with partial impairment of consciousness; (2) the experience of embodiement in virtual reality when the motion of a participant is mapped to the virtual body in real time (Kilteni et al., 2012).

**Figure 1:**
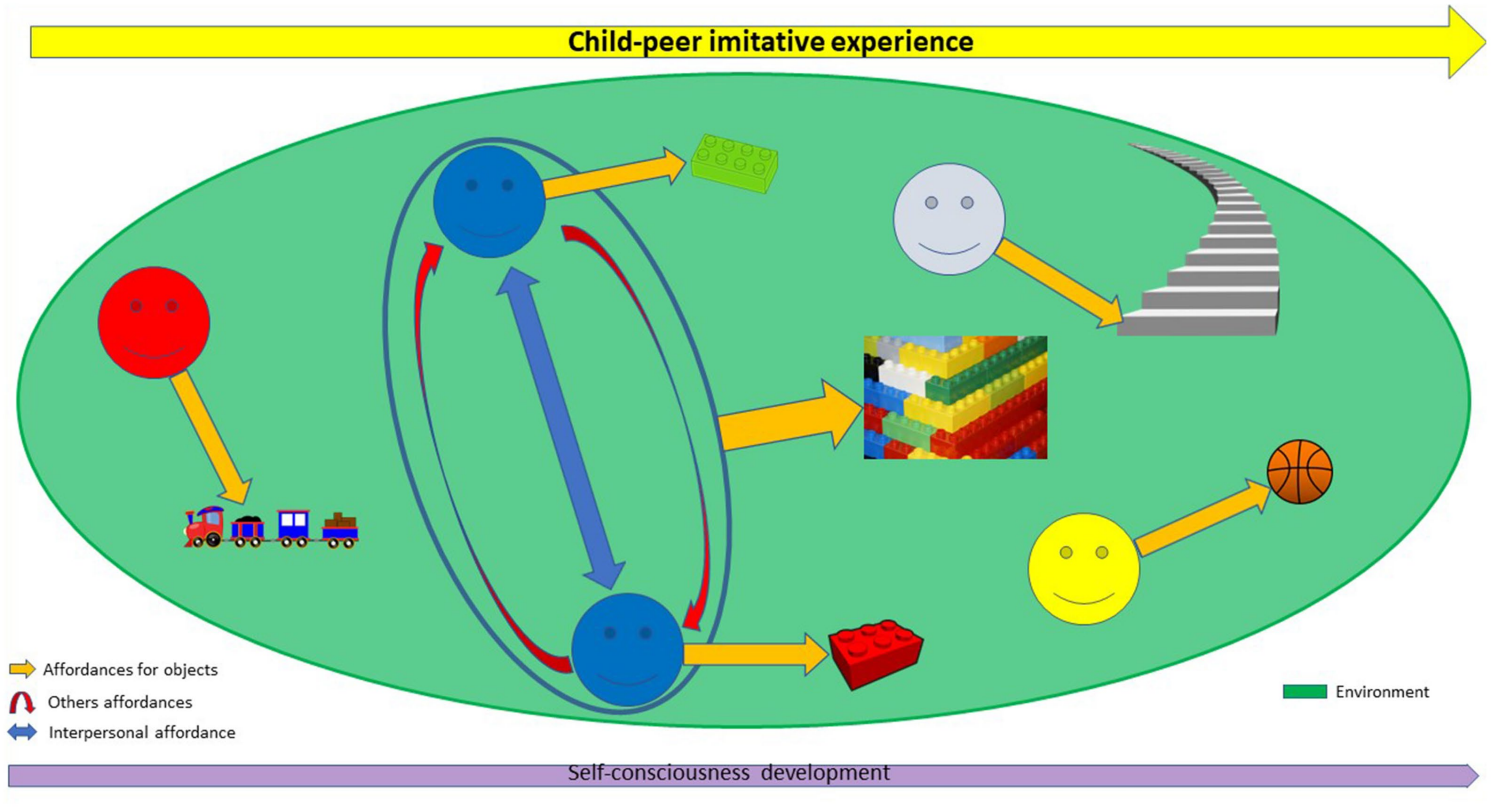
In a developmental perspective, affordance boundaries and self-consciousness from child-peer similarity in imitative experience.

Each child has the ability to perceive self-affordances for objects in the environment. However, according to our hypothesis, child-peer similarity (between the two blue peers) offers the opportunity to engage in child-peer imitation, creating a situation of interpersonal affordance. This situation allowing interpersonal coordination will then direct self-affordances for objects and promote the perception of other’s-affordances (using a lego) for each co-actors. It also enables the emergence of new action possibilities in terms of joint action possibilities, which extends the self-affordances boundaries that are possible for each co-actor (a lego building). Self-consciousness emerges from the imitative experience made possible by the situation of interpersonal affordance between children and their peers (*Cf.* Figure 1).

Finally, this continuously adapting experience, involving shifts between ego- and hetero-centered perspectives, is correlated with the development of executive functions. Aïte et al. (2018) demonstrated that the growing ability to inhibit an egocentric perspective seems to explain the capacity to adopt someone else’s perspective.

To what extent could our hypothesis be applied to peer-mediated interventions in children with autism?

## 5. Motor imitation impairments and peer-mediated interventions for children with autism spectrum disorder

Autism spectrum disorder (ASD) is a neurodevelopmental condition with heterogeneous clinical manifestations including social communication difficulties, behavioral stereotypies and limited interest (American Psychiatric Association, 2013). Its core symptoms are underlined by genetic, environmental and developmental factors (Masini et al., 2020).

Although not cardinal symptoms, motor impairments are fundamental aspects of ASD (Fabbri-Destro et al., 2013; Xavier et al., 2015). Children with ASD can display impairments in control of movements (Frith et al., 2003; Fournier et al., 2010) and in social motor synchrony (Fitzpatrick et al., 2017). In addition, children with ASD show executive dysfunction including difficulties in inhibition (Hill, 2004), while executive functions are linked to imitation abilities (Rogers and Pennington, 1991). Body-self impairments and a relative lack of self-consciousness associated with difficulties in social understanding are also common in this disorder (Tordjman et al., 2019). For Hobson and Meyer (2005), there is a relative failure in the mechanism of self - other connectedness involving the ability ‘to adopt the bodily-anchored psychological and communicative stance of another person’. The development of self-consciousness and the ability to put oneself in another’s shoes are inherent in the imitative process as described above.

Imitation may be a primary deficit in autism that underlies the abnormal development of social-communicative behaviors (Ingersoll, 2008). Children with ASD are known to imitate less frequently and less accurately than TD children (Williams et al., 2004). While children with ASD are able to recognize imitation by others, they appear to be less responsive to being imitated than typical children (Contaldo et al., 2016). This population was found to have similar performances to TD children when imitated actions had a visual goal or meaning but lower performances in copying meaningless (Edwards, 2014) or goal-less imitation tasks (Nielsen et al., 2013). This suggests a failure to use the kinematic details of action (e.g., amplitude, speed, or trajectory; Wild et al., 2010).

A study of the developmental aspects of spontaneous motor imitation in children with ASD, found interpersonal synchronization and motor coordination impairments in children with ASD as compared to TD children and children with developmental coordination disorder (Xavier et al., 2018).

Action vitality forms are highly pervasive aspects of daily life. Their expression and recognition have been widely assumed to be critical for social bonding in children as well as in adults (Trevarthen, 1998; Rochat, 2009). Casartelli et al. (2020) assessed whether and to what extent neurotypical adults understand ASD children performing different actions (placing vs. throwing) with two different vitality forms (rude vs. gentle). The authors found that ASD children express their own vitality forms in a way that is motorically dissimilar to TD children. In addition, they demonstrated that this motor dissimilarity prevents neurotypical adults from recognizing vitality forms, when observing ASD children acting gently or rudely. In sum, beyond the great poorness of the social experience in this population, there would not only be the well-known difficulty to understand the intentions of the others, but also that of typical subjects to understand the motor actions of the autistic child.

According to Gepner and Féron (2009) children with ASD have disabilities to produce real-time sensory-motor coupling, postural adjustments, adequate verbal and nonverbal outputs. This leads the author to highlight the potential usefulness of slowing down the sensory environment for patients with ASD, particularly low functioning, to enhance their imitative abilities.

Hellendoorn et al. (2015) posit that impairments in motor functioning amongst this population are partly explained by an innate deficit in the perception of structural and transformational invariants specifying the action possibilities in the environment. The author hypothesizes that individuals with ASD do not perceive the same affordances as other people do and have difficulties perceiving others’ affordances. Recent studies have reported that children with ASD-ID have more imitation difficulties than children with ASD without intellectual disabilities (Receveur et al., 2005).

Studies of school-aged children with ASD have shown a disrupted pattern of social engagement with fewer reciprocal friendships (Schupp et al., 2013). Peer-mediated interventions (PMI) are effective to address core social communication and social interaction deficits for children with ASD. However, the efficacy of this approach in terms of generalization and sustainment of the improved social skills is underexamined (Chang and Locke, 2016). In PMI, TD peers helping children with ASD are chosen by therapists, parents or teachers because of their strong social skills. They are educated and trained using peer strategies.

In a recent study, Zhang et al. (2022) confirmed the effectiveness of PMI on social skills of children with mild-to-moderate ASD. Conversely, those with lower cognitive functioning could not participate well in the social games proposed.

## 6. Could the ‘similarity hypothesis’ be useful to improve peers interaction in autism?

Some interventions for children with ASD involve the imitation of the child’s actions by therapists or parents, as a strategy to promote social engagement (for a review see Contaldo et al., 2016). These behavioral interventions are mainly used in children at an early age or with low developmental and imitation abilities. Contaldo et al. (2016) conclude that the behavioral similarity between the actions of children and the following action of an adult should be used as a strategy that would deserve to be systematically included in interventions for children with ASD.

For example, reciprocal imitation training (RIT) is a naturalistic imitation intervention developed for young children with autism (Ingersoll, 2008). The goal of RIT is, in a naturalistic setting, to target the two components of imitation that are involved in reciprocal imitation: Imitation production and imitation recognition. The experience of being imitated is critical for the developmental process, and is associated with several social communication skills of children with ASD (Berger and Ingersoll, 2013). RIT notably includes social interactions with an adult which contingently imitates the child’s actions with toys, gestures, body movements and vocalizations.

Using RIT in a more recent study, Berger and Ingersoll (2015) compared how children with ASD and typically developing children respond to being imitated during a naturalistic imitation task. They found that children with ASD engage in all forms of imitation recognition behaviors, even in testing behaviors, however with less frequency than their typically developing peers.

These interventions produce generalized imitation in experimental setting with an adult or in caregiver-child interaction that is maintained in the absence of reinforcement and over time. However, the generalization of imitative behaviors were not assessed in the children’s natural environment or during play with similar aged peers. Future studies addressing generalization to home and school settings as well as the long-term durability of treatment gains are needed.

Several studies, albeit predominantly qualitative, highlighted the power of Dance and Movement Therapy (DMT) in reducing symptoms associated with ASD and increasing communication skills and social development (Mastrominico et al., 2018). Mirroring and rhythm are the key components extensively and consistently used in DMT. Mirroring consists in the imitation by the therapist of the exact shape, form and movement of the patient’s actions, sharing the same body movement or emotional state (Contaldo et al., 2016). Several studies have demonstrated that mirroring is the most effective feature within DMT and a useful tool to enhance communication, outside of DMT (Koch et al., 2015). Rhythm refers to the use of a strong, regular, repeated pattern of movement or sound. Morris et al. (2021) reviewed literature focusing on either mirroring or rhythm in studies that have reliable outcome measures assessing improvements in communication and social skills in ASD children. However, they did not find any study on the association of both. In addition, all involved therapist-child interactions while none investigated their efficacy in child-peer interactions.

Our general hypothesis is that child-peer similarity fosters ‘imitability’ (*cf.* 4) and, consequently, facilitates subsequent cognitive processing including inhibition abilities. We argue that, in PMI, an encounter between children with autism (particularly those with lower cognitive functioning) and similar peers would offer the opportunity to engage in a spontaneous process of interpersonal coordination. This matching will be organized based on interpersonal morphological, behavioral and motor similarity (*Cf.* 4).

The contiguity between oneself and others, through a proprioceptive and kinesthesic matching, could facilitate behavioral mirroring and interpersonal synchronization.

Such a tailored environment, through behavior copying, provides the conditions for a spontaneous dynamic interpersonal coordination process leading to the development of self-consciousness and the emergence of perception of interpersonal, self and others’ affordances. During child development, the continuous congruence between one’s own body size and motor ability evolution, and that of others, would sustain a generalization of peer-interactive experiences favoring adaptation for children with autism.

## 7. Conclusion

Our general hypothesis resonates well with the idea, carried by clinical insights and intersubjective theories, that there is a need for a relational and interactional shift in the research in autism (Bolis and Schilbach, 2018). The aim of future studies could be to test our hypothesis on children with autism versus TD children or between children with autism. They could be used first in an observational study in line with Nadel (2002) work: spontaneous imitation between children with autism could then be recorded and analysed using these metrics.

If this hypothesis is confirmed, this similarity should be considered when caring for ASD children, specifically regarding PMI. In a future study, we could test the comparison between two groups of children in PMI [(i) children with ASD matched with TD children on the basis of this similarity and (ii) children with ASD and TD children, i.e., PMI as usual]. The purpose would be to demonstrate a greater improvement of the communication and social skills, using behavioral measures as imitation, communication, social reciprocity, and joint attention (in line with the study of Ingersoll et al., 2012), in children with ASD in the first group.

## Data availability statement

The original contributions presented in the study are included in the article/supplementary material, further inquiries can be directed to the corresponding author.

## Author contributions

JX, SJ, and DC contributed to the writing and proofreading of the manuscript they provide approval for publication of the content. All authors contributed to the article and approved the submitted version.

## Conflict of interest

The authors declare that the research was conducted in the absence of any commercial or financial relationships that could be construed as a potential conflict of interest.

## Publisher’s note

All claims expressed in this article are solely those of the authors and do not necessarily represent those of their affiliated organizations, or those of the publisher, the editors and the reviewers. Any product that may be evaluated in this article, or claim that may be made by its manufacturer, is not guaranteed or endorsed by the publisher.

## References

[ref1] Agnetta B. Rochat P. (2004). Imitative games by 9-, 14-, and 18-month-old infants. Infancy 6, 1–36. doi: 10.1207/s15327078in0601

[ref2] Aïte A. Cassotti M. Linzarini A. Osmont A. Houdé O. Borst G. (2018). Adolescents’ inhibitory control: keep it cool or lose control. Dev. Sci. 21:e12491. doi: 10.1111/desc.12491 27882631

[ref3] American Psychiatric Association (2013). Diagnostic and Statistical Manual of Mental Disorders: DSM-5. Arlington, TX: American Psychiatric Publishing Incorporated

[ref4] Bardy B. G. Salesse R. N. Gueugnon M. Zhong Z. Lagarde J. Marin L. (2014). “Movement similarities and differences during social interaction: the scientific foundation of the ALTEREGO European project” in 2014 IEEE International Conference on Systems, Man, and Cybernetics (SMC). Presented at the 2014 IEEE International Conference on Systems, Man and Cybernetics - SMC (San Diego, CA, USA: IEEE), 772–777.

[ref5] Berger N. I. Ingersoll B. (2013). An exploration of imitation recognition in young children with autism spectrum disorders. Autism Res. 6, 411–416. doi: 10.1002/aur.1303, PMID: 23696180

[ref9001] Berger N. Ingersoll B. (2015). An evaluation of imitation recognition abilities in typically developing children and young children with autism spectrum disorder. Autism Res. 8, 442–453. doi: 10.1002/aur.1462 25707498

[ref6] Blanke O. Mohr C. (2005). Out-of-body experience, heautoscopy, and autoscopic hallucination of neurological origin implications for neurocognitive mechanisms of corporeal awareness and self-consciousness. Brain Res. Brain Res. Rev. 50, 184–199. doi: 10.1016/j.brainresrev.2005.05.008, PMID: 16019077

[ref7] Bolis D. Schilbach L. (2018). Observing and participating in social interactions: action perception and action control across the autistic spectrum. Dev. Cogn. Neurosci. 29, 168–175. doi: 10.1016/j.dcn.2017.01.009, PMID: 28188104 PMC6987847

[ref9] Byrne R. (2009). Animal imitation. Curr. Biol. 19, R111–R114. doi: 10.1016/j.cub.2008.11.027 19211046

[ref10] Casartelli L. Federici A. Fumagalli L. Cesareo A. Nicoli M. Ronconi L.. (2020). Neurotypical individuals fail to understand action vitality form in children with autism spectrum disorder. Proc. Natl. Acad. Sci. U. S. A. 117, 27712–27718. doi: 10.1073/pnas.2011311117, PMID: 33087573 PMC7959533

[ref11] Chang Y.-C. Locke J. (2016). A systematic review of peer-mediated interventions for children with autism spectrum disorder. Res. Autism Spectr. Disord. 27, 1–10. doi: 10.1016/j.rasd.2016.03.010, PMID: 27807466 PMC5087797

[ref12] Contaldo A. Colombi C. Narzisi A. Muratori F. (2016). The social effect of “being imitated” in children with autism Spectrum disorder. Front. Psychol. 7:7. doi: 10.3389/fpsyg.2016.00726 27242632 PMC4865518

[ref13] Costantini M. Committeri G. Sinigaglia C. (2011). Ready both to your and to my hands: mapping the action space of others. PLoS One 6:e17923. doi: 10.1371/journal.pone.0017923, PMID: 21483734 PMC3070696

[ref14] Creem-Regehr S. Gagnon K. Geuss M. Stefanucci J. (2013). Relating spatial perspective taking to the perception of other’s affordances: providing a foundation for predicting the future behavior of others. Front. Hum. Neurosci 7:7. doi: 10.3389/fnhum.2013.00596 24068992 PMC3781345

[ref15] de Waal F. B. M. (2008). Putting the altruism Back into altruism: the evolution of empathy. Annu. Rev. Psychol. 59, 279–300. doi: 10.1146/annurev.psych.59.103006.093625, PMID: 17550343

[ref16] Dijk L. Rietveld E. (2017). Foregrounding sociomaterial practice in our understanding of affordances: the skilled intentionality framework. Front. Psychol. 7:1969. doi: 10.3389/fpsyg.2016.01969, PMID: 28119638 PMC5220071

[ref9002] Eckerman C. Didow S. (1996). Nonverbal imitation and toddlers’ mastery of verbal means of achieving coordinated action. Dev. Psychol. 32, 141–152.

[ref18] Edwards L. A. (2014). A meta-analysis of imitation abilities in individuals with autism spectrum disorders. Autism Res 7, 363–380. doi: 10.1002/aur.1379, PMID: 24863681

[ref19] Erksine R. G. (1998). Attunement and involvement: therapeutic responses to relational needs. Int. J. Psychother. 3:13.

[ref20] Fabbri-Destro M. Gizzonio V. Avanzini P. (2013). Autism, motor dysfunctions and mirror mechanism. Clin. Neuropsychiatry 10, 177–187.

[ref21] Fitzpatrick P. Romero V. Amaral J. Duncan A. Barnard H. Richardson M.. (2017). Evaluating the importance of social motor synchronization and motor skill for understanding autism. Autism Res. 10, 1687–1699. doi: 10.1002/aur.1808, PMID: 28590041 PMC5648610

[ref22] Fletcher-Watson S. Findlay J. M. Leekam S. R. Benson V. (2008). Rapid detection of person information in a naturalistic scene. Perception 37, 571–583. doi: 10.1068/p5705, PMID: 18546664

[ref23] Fournier K. A. Hass C. J. Naik S. K. Lodha N. Cauraugh J. H. (2010). Motor coordination in autism Spectrum disorders: a synthesis and meta-analysis. J. Autism Dev. Disord. 40, 1227–1240. doi: 10.1007/s10803-010-0981-3, PMID: 20195737

[ref24] Franchak J. M. (2019). Development of affordance perception and recalibration in children and adults. J. Exp. Child Psychol. 183, 100–114. doi: 10.1016/j.jecp.2019.01.016, PMID: 30870696

[ref25] Frith U. Hill E. L. Mari M. Castiello U. Marks D. Marraffa C.. (2003). The reach–to–grasp movement in children with autism spectrum disorder. Philos. Trans. R. Soc. Lond. Ser. B Biol. Sci. 358, 393–403. doi: 10.1098/rstb.2002.1205, PMID: 12639336 PMC1693116

[ref27] Gagnon D. Na K. Heiner J. Stefanucci S. Creem-Regehr B. Bodenheimer. (2020). The Role of Viewing Distance and Feedback on Affordance Judgments in Augmented Reality, In: 2020 IEEE Conference on Virtual Reality and 3D User Interfaces (VR). Presented at the 2020 IEEE Conference on Virtual Reality and 3D User Interfaces (VR), pp. 922–929

[ref30] Gepner B. Féron F. (2009). Autism: a world changing too fast for a mis-wired brain? Neurosci. Biobehav. Rev. Special Section: Neuroscience and Biobehavioral Research: A Chinese Perspective 33, 1227–1242. doi: 10.1016/j.neubiorev.2009.06.006, PMID: 19559043

[ref31] Gibson J. J. (1979). The Ecological Approach to Visual Perception. Houghton, Mifflin and Company, Boston, MA, USA.

[ref32] Hellendoorn A. Wijnroks L. Leseman P. P. M. (2015). Unraveling the nature of autism: finding order amid change. Front. Psychol. 6:6. doi: 10.3389/fpsyg.2015.00359 25870581 PMC4378365

[ref33] Hill E. L. (2004). Executive dysfunction in autism. Trends Cogn. Sci. 8, 26–32. doi: 10.1016/j.tics.2003.11.003 14697400

[ref34] Hobson R. P. Meyer J. A. (2005). Foundations for self and other: a study in autism. Dev. Sci. 8, 481–491. doi: 10.1111/j.1467-7687.2005.00439.x, PMID: 16246239

[ref9003] Ingersoll B. (2012). Brief report: effect of a focused imitation intervention on social functioning in children with autism. J. Autism Dev. Disord. 42, 1768–1773. doi: 10.1007/s10803-011-1423-6 22146934 PMC3667953

[ref35] Ingersoll B. (2008). The social role of imitation in autism: implications for the treatment of imitation deficits. Infants Young Child. 21, 107–119. doi: 10.1097/01.IYC.0000314482.24087.14

[ref36] Keromnes G. Chokron S. Celume M.-P. Berthoz A. Botbol M. Canitano R.. (2019). Exploring self-consciousness from self- and other-image recognition in the Mirror: concepts and evaluation. Front. Psychol. 10:719. doi: 10.3389/fpsyg.2019.00719, PMID: 31133909 PMC6524719

[ref37] Kilner J. Hamilton A. F. D. C. Blakemore S. J. (2007). Interference effect of observed human movement on action is due to velocity profile of biological motion. Soc. Neurosci. 2, 158–166. doi: 10.1080/17470910701428190, PMID: 18633814

[ref38] Kilteni K. Normand J.-M. Sanchez-Vives M. V. Slater M. (2012). Extending body space in immersive virtual reality: a very long arm illusion. PLoS One 7:e40867. doi: 10.1371/journal.pone.0040867, PMID: 22829891 PMC3400672

[ref39] Kimmel M. Hristova D. Kussmaul K. (2018). Sources of embodied creativity: interactivity and ideation in contact improvisation. Behav. Sci. Basel Switz. 8:52. doi: 10.3390/bs8060052, PMID: 29882858 PMC6027199

[ref9004] Koch S. C. Mehl L. Sobanski E. Sieber M. Fuchs T. (2015). Fixing the mirrors: A feasibility study of the effects of dance movement therapy on young adults with autism spectrum disorder. Autism. 19, 338–350. doi: 10.1177/1362361314522353 24566716

[ref40] Kronsted C. Gallagher S. (2021). Dances and affordances: the relationship between dance training and conceptual problem-solving. J. Aesthet. Educ. 55, 35–55. doi: 10.5406/jaesteduc.55.1.0035

[ref43] Leclère C. Viaux S. Avril M. Achard C. Chetouani M. Missonnier S.. (2014). Why synchrony matters during mother-child interactions: a systematic review. PLoS One 9:e113571. doi: 10.1371/journal.pone.0113571, PMID: 25469637 PMC4254467

[ref44] Liberman Z. Woodward A. L. Kinzler K. D. (2016). Preverbal infants infer third party relationships based on language. Cogn. Sci. 41 Suppl 3, 622–634. doi: 10.1111/cogs.12403, PMID: 27471173 PMC6139255

[ref45] Lopresti-Goodman S. M. Richardson M. J. Baron R. M. Carello C. Marsh K. L. (2009). Task constraints on affordance boundaries. Mot. Control. 13, 69–83. doi: 10.1123/mcj.13.1.69, PMID: 19246779

[ref46] Mark L. Nemeth K. Gardner D. Dainoff M. Paasche J. Duffy M.. (1997). Postural dynamics and the preferred critical boundary for visually guided reaching. J. Exp. Psychol. Hum. Percept. Perform. 23, 1365–1379. doi: 10.1037/0096-1523.23.5.1365, PMID: 9336957

[ref47] Marsh K. L. Richardson M. J. Baron R. M. Schmidt R. C. (2006). Contrasting approaches to perceiving and acting with others. Ecol. Psychol. 18, 1–38. doi: 10.1207/s15326969eco1801_1

[ref48] Masini E. Loi E. Vega-Benedetti A. F. Carta M. Doneddu G. Fadda R.. (2020). An overview of the Main genetic, epigenetic and environmental factors involved in autism Spectrum disorder focusing on synaptic activity. Int. J. Mol. Sci. 21:8290. doi: 10.3390/ijms21218290, PMID: 33167418 PMC7663950

[ref49] Mastrominico A. Fuchs T. Manders E. Steffinger L. Hirjak D. Sieber M.. (2018). Effects of dance movement therapy on adult patients with autism Spectrum disorder: a randomized controlled trial. Behav. Sci. Basel Switz. 8:61. doi: 10.3390/bs8070061, PMID: 29966313 PMC6071290

[ref51] Meltzoff A. N. (2007). ‘Like me’: a foundation for social cognition. Dev. Sci. 10, 126–134. doi: 10.1111/j.1467-7687.2007.00574.x, PMID: 17181710 PMC1852489

[ref50] Meltzoff A. N. (1990). “Foundations for developing a concept of self: the role of imitation in relating self to other and the value of social mirroring, social modeling, and self practice in infancy” in The Self in Transition: Infancy to Childhood. eds. Cicchetti D. Beeghly M. (Chicago, IL, US: University of Chicago Press), 139–164.

[ref52] Meltzoff A. N. Decety J. (2003). What imitation tells us about social cognition: a rapprochement between developmental psychology and cognitive neuroscience. Philos. Trans. R. Soc. Lond. Ser. B Biol. Sci. 358, 491–500. doi: 10.1098/rstb.2002.1261, PMID: 12689375 PMC1351349

[ref54] Morris P. Hope E. Foulsham T. Mills J. P. (2021). The effectiveness of mirroring- and rhythm-based interventions for children with autism Spectrum disorder: a systematic review. Rev. J. Autism Dev. Disord. 8, 541–561. doi: 10.1007/s40489-021-00236-z

[ref57] Nadel-Brulfert J. Baudonniere P. M. (1982). The social function of reciprocal imitation in 2-year-old peers. Int. J. Behav. Dev. 5, 95–109. doi: 10.1177/016502548200500105

[ref55] Nadel J. (1986). Imitation et Communication Entre Jeunes Enfants, Croissance de L’enfant Genèse de L’homme. Presses Universitaires de France Paris Cedex 14.

[ref56] Nadel J. (2002). “Imitation and imitation recognition: functional use in preverbal infants and nonverbal children with autism” in The Imitative Mind: Development, Evolution, and Brain Bases, Cambridge Studies in Cognitive Perceptual Development (New York, NY, US: Cambridge University Press), 42–62.

[ref58] Nielsen M. (2006). Copying actions and copying outcomes: social learning through the second year. Dev. Psychol. 42, 555–565. doi: 10.1037/0012-1649.42.3.555, PMID: 16756445

[ref9005] Nielsen M. Slaughter V. Dissanayake C. (2013). Object-directed imitation in children with high-functioning autism: testing the social motivation hypothesis: autism, overimitation, and synchronic imitation. Autism Res. 6, 23–32. doi: 10.1002/aur.1261 23166017

[ref59] Powell L. J. Spelke E. S. (2013). Preverbal infants expect members of social groups to act alike. Proc. Natl. Acad. Sci. U. S. A. 110, E3965–E3972. [PubMed: 24062446]. doi: 10.1073/pnas.1304326110, PMID: 24062446 PMC3799333

[ref60] Receveur C. Lenoir P. Desombre H. Roux S. Barthelemy C. Malvy J. (2005). Interaction and imitation deficits from infancy to 4 years of age in children with autism: a pilot study based on videotapes. Autism 9, 69–82. doi: 10.1177/1362361305049030, PMID: 15618263

[ref61] Rochat P. (1995). Perceived reachability for self and for others by 3- to 5-year-old children and adults. J. Exp. Child Psychol. 59, 317–333. doi: 10.1006/jecp.1995.1014, PMID: 7722438

[ref62] Rochat P. (2009). Others in Mind: Social Origins of Self-Consciousness Cambridge University Press.

[ref63] Rochat P. Zahavi D. (2011). The uncanny mirror: a re-framing of mirror self-experience. Conscious. Cogn. 20, 204–213. doi: 10.1016/j.concog.2010.06.007, PMID: 20889353

[ref64] Rogers S. J. Pennington B. F. (1991). A theoretical approach to the deficits in infantile autism. Dev. Psychopathol. 3, 137–162. doi: 10.1017/S0954579400000043

[ref65] Romero V. Coey C. Schmidt R. C. Richardson M. J. (2012). Movement coordination or movement interference: visual tracking and spontaneous coordination modulate rhythmic movement interference. PLoS One 7, 1–9. doi: 10.1371/journal.pone.0044761, PMID: 23028607 PMC3444463

[ref66] Schupp C. W. Simon D. Corbett B. A. (2013). Cortisol responsivity differences in children with autism spectrum disorders during free and cooperative play. J. Autism Dev. Disord. 43, 2405–2417. doi: 10.1007/s10803-013-1790-2, PMID: 23430177 PMC3885342

[ref67] Słowiński P. Zhai C. Alderisio F. Salesse R. Gueugnon M. Marin L.. (2016). Dynamic similarity promotes interpersonal coordination in joint action. J. R. Soc. Interface 13:20151093. doi: 10.1098/rsif.2015.1093, PMID: 27009178 PMC4843673

[ref68] Steffensen S. V. (2013). “Human interactivity: problem-solving, solution-probing and verbal patterns in the Wild” in Cognition beyond the brain: Computation, interactivity and human artifice. eds. Cowley S. J. Vallée-Tourangeau F. (London: Springer), 195–221. doi: 10.1007/978-1-4471-5125-8_11

[ref69] Stoffregen T. A. Smart L. J. Bardy B. G. Pagulayan R. J. (1999). Postural stabilization of looking. J. Exp. Psychol. Hum. Percept. Perform. 25, 1641–1658. doi: 10.1037/0096-1523.25.6.1641

[ref70] Tanaka S. (2015). Intercorporeality as a theory of social cognition. Theory Psychol. 25, 455–472. doi: 10.1177/0959354315583035, PMID: 28458467 PMC5390942

[ref71] Tomasello M. (1990). “Cultural transmission in the tool use and communicatory signaling of chimpanzees?” in “Language” and intelligence in monkeys and apes: Comparative developmental perspectives. eds. Gibson K. R. Parker S. T. (Cambridge: Cambridge University Press), 274–311. doi: 10.1017/CBO9780511665486.012

[ref72] Tomasello M. (1999). “Having intentions, understanding intentions, and understanding communicative intentions” in Developing theories of intention. eds. Zelazo P. D. Astington J. W. Olson D. R. (Hillsdale, NJ: Erlbaum), 63–75.

[ref73] Tomassini A. Laroche J. Emanuele M. Nazzaro G. Petrone N. Fadiga L.. (2022). Interpersonal Synchronization of Movement Intermittency. iScience 25:25. doi: 10.1016/j.isci.2022.104096, PMID: 35372806 PMC8971945

[ref74] Tordjman S. Celume M. P. Denis L. Motillon T. Keromnes G. (2019). Reframing schizophrenia and autism as bodily self-consciousness disorders leading to a deficit of theory of mind and empathy with social communication impairments. Neurosci. Biobehav. Rev. 103, 401–413. doi: 10.1016/j.neubiorev.2019.04.007, PMID: 31029711

[ref75] Trees A. R. (2009). “Interactional sensitivity: rating social support, attachment, and interaction in adult relationships” in The sourcebook of nonverbal measures: Going beyondwords. ed. Manusov V. (New York, NY: Routledge), 251–266.

[ref76] Trevarthen C. (1998) in The Concept and Foundations of Infant Intersubjectivity in Intersubjective Communication and Emotion in Early Ontogeny. ed. Bråten S. (Cambridge University Press), 15–46.

[ref77] Varela F. J. Rosch E. Thompson E. (1991). The Embodied Mind: Cognitive Science and Human Experience. Cambridge, MA: The MIT Press.

[ref80] Wallon H. (1949) Les Origines du Caractère Chez L’enfant, Paris, P.U.F.

[ref81] Wallon H. (1956). Levels and fluctuations of the ego. Evol. Psychiatr., 389–401. PMID: 13330769

[ref82] Warren W. H. Jr. (1998). “The state of flow” in High-Level Motion Processing: Computational, Neurobiological, and Psychophysical Perspectives (Cambridge, MA, USA: The MIT Press), 315–358.

[ref83] Warren W. H. Whang S. (1987). Visual guidance of walking through apertures: body-scaled information for affordances. J. Exp. Psychol. Hum. Percept. Perform. 13, 371–383. doi: 10.1037//0096-1523.13.3.371, PMID: 2958586

[ref84] Wild K. Poliakoff E. Gowen E. (2010). The influence of goals on movement kinematics during imitation. Exp Brain Res 204, 353–360. doi: 10.1007/s00221-009-2034-8 19826797

[ref86] Williams J. H. G. Whiten A. Singh T. (2004). A systematic review of action imitation in autistic spectrum disorder. J. Autism Dev. Disord. 34, 285–299. doi: 10.1023/b:jadd.0000029551.56735.3a, PMID: 15264497

[ref87] Xavier J. Bursztejn C. Stiskin M. Canitano R. Cohen D. (2015). Autism spectrum disorders: an historical synthesis and a multidimensional assessment toward a tailored therapeutic program. Res. Autism Spectr. Disord. 18, 21–33. doi: 10.1016/j.rasd.2015.06.011

[ref88] Xavier J. Gauthier S. Cohen D. Zahoui M. Chetouani M. Villa F.. (2018). Interpersonal synchronization, motor coordination, and control are impaired during a dynamic imitation task in children with autism Spectrum disorder. Front. Psychol. 9:1467. doi: 10.3389/fpsyg.2018.01467, PMID: 30233439 PMC6129607

[ref89] Xavier J. Magnat J. Sherman A. Gauthier S. Cohen D. Chaby L. (2016). A developmental and clinical perspective of rhythmic interpersonal coordination: from mimicry toward the interconnection of minds. J. Physiol. Paris 110, 420–426. doi: 10.1016/j.jphysparis.2017.06.001, PMID: 28625683

[ref90] Xavier J. Tilmont E. Bonnot O. (2013). Children’s synchrony and rhythmicity in imitation of peers: toward a developmental model of empathy. J. Physiol. Paris 107, 291–297. doi: 10.1016/j.jphysparis.2013.03.012 23583461

[ref91] Zhang B. Liang S. Chen J. Chen L. Chen W. Tu S.. (2022). Effectiveness of peer-mediated intervention on social skills for children with autism spectrum disorder: a randomized controlled trial. Transl. Pediatr. 11, 663–675. doi: 10.21037/tp-22-110, PMID: 35685075 PMC9173870

